# Supplementary material for: From sequence to enzyme mechanism using multi-label machine learning
Source: BMC Bioinformatics. 2014 May 19;15:150. doi: 10.1186/1471-2105-15-150 (PMC4229970; doi:10.1186/1471-2105-15-150)
Supplement: Additional file 2 — Java code of ml2db. Additional file ml2db_code.tar.gz contains the Java source code to run the multi-label machine learning experiments and save the results to database. The code’s Javadoc is included. [file 1471-2105-15-150-S2.zip › additional file 2/ml2db/ml2db/doc/mulan/classifier/lazy/IBLR_ML.html]

IBLR\_ML


JavaScript is disabled on your browser.


- Overview
- Package
- Class
- Use
- Tree
- Deprecated
- Index
- Help

- Prev Class
- Next Class

- Frames
- No Frames

- All Classes

- Summary:
- Nested |
- Field |
- Constr |
- Method

- Detail:
- Field |
- Constr |
- Method


mulan.classifier.lazy

## Class IBLR\_ML

- java.lang.Object
- - mulan.classifier.MultiLabelLearnerBase
  - - mulan.classifier.lazy.MultiLabelKNN
    - - mulan.classifier.lazy.IBLR\_ML

- All Implemented Interfaces:
  :   java.io.Serializable, MultiLabelLearner, weka.core.TechnicalInformationHandler

  ---

    

  ```
  public class IBLR_ML
  extends MultiLabelKNN
  ```

  This class is an implementation of the "IBLR-ML" and "IBLR-ML+" methods for the MULAN package.

  Version:
  :   2010.12.29

  Author:
  :   Weiwei Cheng, Eleftherios Spyromitros-Xioufis, Grigorios Tsoumakas

  See Also:
  :   Serialized Form

- - ### Field Summary

    - ### Fields inherited from class mulan.classifier.lazy.MultiLabelKNN

      `WEIGHT_INVERSE, WEIGHT_NONE, WEIGHT_SIMILARITY`
  - ### Constructor Summary

    Constructors

    Constructor and Description

    `IBLR_ML()`

    Default constructor uses 10 NN

    `IBLR_ML(int numNeighbors)`

    Constructor that sets the number of neighbors

    `IBLR_ML(int numNeighbors,
    boolean addFeatures)`

    Full constructor
  - ### Method Summary

    Methods

    | Modifier and Type | Method and Description |
    | --- | --- |
    | `weka.core.TechnicalInformation` | `getTechnicalInformation()` Returns an instance of a TechnicalInformation object, containing detailed information about the technical background of this class, e.g., paper reference or book this class is based on. |
    | `java.lang.String` | `globalInfo()` Returns a string describing the multi-label learner. |

    - ### Methods inherited from class mulan.classifier.lazy.MultiLabelKNN

      `isUpdatable, setDfunc, setDistanceWeighting`
    - ### Methods inherited from class mulan.classifier.MultiLabelLearnerBase

      `build, getDebug, makeCopy, makePrediction, setDebug`
    - ### Methods inherited from class java.lang.Object

      `equals, getClass, hashCode, notify, notifyAll, toString, wait, wait, wait`

- - ### Constructor Detail


    - #### IBLR\_ML

      ```
      public IBLR_ML()
      ```

      Default constructor uses 10 NN


    - #### IBLR\_ML

      ```
      public IBLR_ML(int numNeighbors)
      ```

      Constructor that sets the number of neighbors

      Parameters:
      :   `numNeighbors` - the number of nearest neighbors considered


    - #### IBLR\_ML

      ```
      public IBLR_ML(int numNeighbors,
             boolean addFeatures)
      ```

      Full constructor

      Parameters:
      :   `numNeighbors` - the number of nearest neighbors considered
      :   `addFeatures` - when true, IBLR-ML+ is used
  - ### Method Detail


    - #### globalInfo

      ```
      public java.lang.String globalInfo()
      ```

      **Description copied from class: `MultiLabelLearnerBase`**

      Returns a string describing the multi-label learner.

      **Specified by:**
      :   `globalInfo` in class `MultiLabelLearnerBase`

      Returns:
      :   a description suitable for displaying in a future gui


    - #### getTechnicalInformation

      ```
      public weka.core.TechnicalInformation getTechnicalInformation()
      ```

      **Description copied from class: `MultiLabelLearnerBase`**

      Returns an instance of a TechnicalInformation object, containing detailed
      information about the technical background of this class, e.g., paper
      reference or book this class is based on.

      **Specified by:**
      :   `getTechnicalInformation` in interface `weka.core.TechnicalInformationHandler`

      **Specified by:**
      :   `getTechnicalInformation` in class `MultiLabelLearnerBase`

      Returns:
      :   the technical information about this class


- Overview
- Package
- Class
- Use
- Tree
- Deprecated
- Index
- Help

- Prev Class
- Next Class

- Frames
- No Frames

- All Classes

- Summary:
- Nested |
- Field |
- Constr |
- Method

- Detail:
- Field |
- Constr |
- Method
